# Acute phlegmonous appendicitis with deciduosis: a case report and literature review

**DOI:** 10.1093/jscr/rjaf375

**Published:** 2025-06-05

**Authors:** Takuya Shimogawa, Takanobu Yamao, Nobuya Daitoku, Mayumi Nagayasu, Hiroyuki Koita, Kunitaka Kuramoto

**Affiliations:** Department of Gastroenterological Surgery, Graduate School of Medical Sciences Kumamoto University, 1-1-1 Honjo, Kumamoto, 860-8556, Japan; Department of Surgery, National Hospital Organization Miyakonojo Medical Center, 5033-1 Iwayoshi-cho, Miyakonojo, Miyazaki, 885-0014, Japan; Department of Surgery, National Hospital Organization Miyakonojo Medical Center, 5033-1 Iwayoshi-cho, Miyakonojo, Miyazaki, 885-0014, Japan; Department of Surgery, National Hospital Organization Miyakonojo Medical Center, 5033-1 Iwayoshi-cho, Miyakonojo, Miyazaki, 885-0014, Japan; Department of Diagnostic Pathology, National Hospital Organization Miyakonojo Medical Center, 5033-1 Iwayoshi-cho, Miyakonojo, Miyazaki, 885-0014, Japan; Department of Diagnostic Pathology, National Hospital Organization Miyakonojo Medical Center, 5033-1 Iwayoshi-cho, Miyakonojo, Miyazaki, 885-0014, Japan; Department of Surgery, National Hospital Organization Miyakonojo Medical Center, 5033-1 Iwayoshi-cho, Miyakonojo, Miyazaki, 885-0014, Japan

**Keywords:** acute appendicitis, decidual membrane, decidualization, appendiceal endometriosis, emergency surgery

## Abstract

Acute appendicitis is a common disease in pregnancy that is often difficult to diagnose. However, delayed diagnosis may have a negative impact on the pregnant woman and the fetus. In this report, we present the case of a 34-year-old pregnant woman with acute appendicitis. The appendicitis was diagnosed based on her abdominal symptoms, but it was difficult to diagnose based on preoperative imaging studies. Pathological examination led to the diagnosis of appendiceal decidua. Acute appendicitis due to appendiceal decidua is extremely rare and has only been described in a few case reports. It is difficult to diagnose appendiceal decidua from imaging findings. When a pregnant woman is diagnosed with acute abdomen, clinicians should consider appendiceal decidua as one of the causes of acute appendicitis.

## Introduction

Acute appendicitis is one of the most common causes of acute abdomen and is also common in pregnancy [1]. However, the physiological and anatomical changes associated with pregnancy may obscure the diagnosis of appendicitis. Furthermore, a delay in diagnosing appendicitis during pregnancy and a delay until surgical intervention may lead to fetal and/or maternal morbidity or mortality [2]. A common cause of appendicitis is obstruction of the appendix lumen by a fecalith (calcified fecal deposit), foreign body, or tumor [3], while acute appendicitis caused by appendiceal decidua is rare. Appendiceal decidua refers to ectopic decidua found in the appendix; ectopic decidua is also known as deciduosis. This differs from the decidual membrane, which is the tissue that forms in the uterine lining during pregnancy.

We report a 34-year-old woman who developed acute phlegmonous appendicitis with deciduosis. In previous case reports, appendiceal decidua has been confused with appendiceal endometriosis. Herein, we describe the differences between the two diseases and review previous case reports.

## Case report

**Figure 1:**
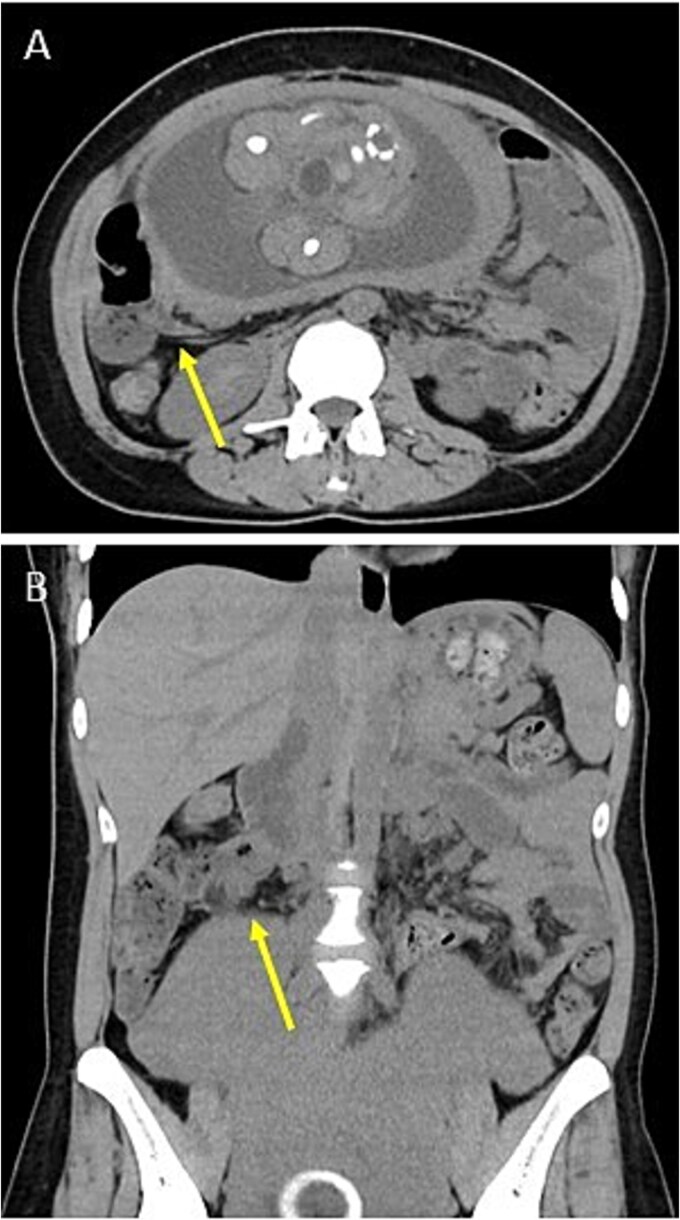
Preoperative images. Plain CT shows no appendix in the axial plane (A) or the coronal plane (B). Arrows indicate the terminal ileum.

A 34-year-old woman (gravida 3, para 1) presented at 28 weeks of gestation with a chief complaint of right abdominal pain that began in the morning. She had no history of gynecological problems. Blood tests showed an increased white blood cell (WBC) count of 17 080/μl and C-reactive protein (CRP) concentration of 3.01 mg/dl. Abdominal ultrasonography revealed a small amount of ascites on the right side of the abdomen; however, the appendix could not be detected. Similarly, an abdominal computed tomography (CT) scan did not reveal the appendix (Fig. 1), and gynecological diseases were ruled out. However, as her abdominal pain worsened in the evening, we diagnosed her with acute appendicitis and decided to perform surgery that night.

**Figure 2:**
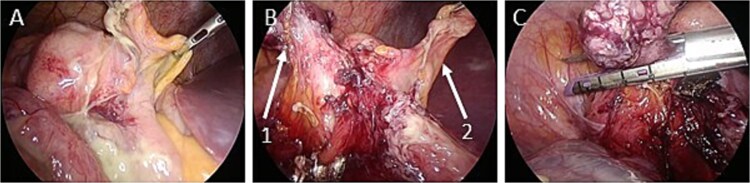
Intraoperative images. (A) There is contaminated ascites around the ileocecal area. (B) The appendiceal mesentery and appendiceal artery are dissected (arrow 1: appendix; arrow 2: ileocecal fold). (C) The appendix is dissected with an automatic suture device.

The operation was performed laparoscopically. The ileocecal area was compressed in the cephalad region due to an enlarged uterus. Contaminated ascites was observed around the ileocecal area (Fig. 2A). An enlarged appendix was identified dorsal to the ileocecal area. The appendix was resected using an automatic suture device (Fig. 2B and C).

**Figure 3:**
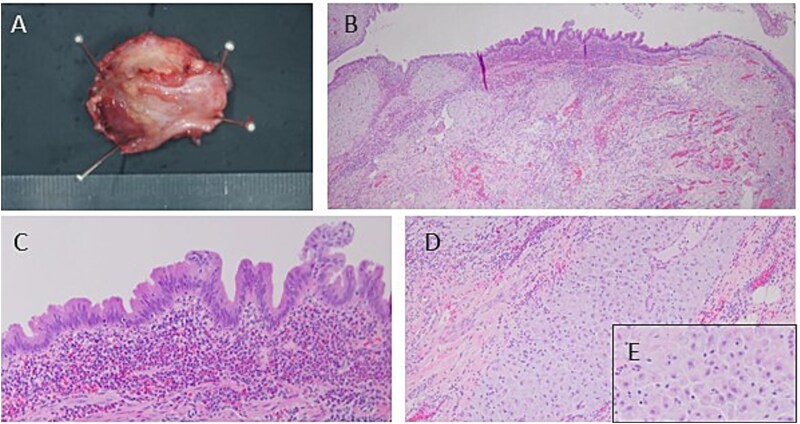
Image of the resected specimen. (A) The resected appendix has a thickened appendix wall and a narrowed appendix lumen. (B–E) Microscopically, there is neutrophilic infiltration on the mucosal side of the appendix and decidual membrane in the stroma.

Microscopic examination of the resected specimen revealed neutrophilic infiltration from the appendiceal mucosa to the subserosa, resulting in a definitive diagnosis of acute appendicitis. In addition, the appendiceal wall stroma contained cells with enlarged nuclei and pale, acidophilic cytoplasm, indicating the presence of decidual membrane. However, no obvious endometrial glandular structures were observed (Fig. 3). Based on these results, we diagnosed the patient with acute phlegmonous appendicitis with deciduosis.

She was discharged home on postoperative day 7. On postoperative day 64 (at 37 weeks gestation), she delivered a 3154 g baby boy by cesarean section due to placenta previa. Both mother and baby have been healthy since then. At the 1-month postoperative follow-up, no complications such as recurrent abdominal pain or surgical site infection were identified, and follow-up was concluded.

## Discussion

We report a rare case of acute phlegmonous appendicitis with deciduosis. Appendicitis is the most common cause of acute abdomen in pregnancy, occurring in approximately one in 1500 deliveries [1]. The prevalence of appendiceal endometriosis is estimated to be 0.7% among women who have undergone emergency appendectomy [4], but the prevalence of appendiceal decidua is unknown. Extrauterine or ectopic decidua is most commonly seen in the ovaries, cervix, uterine serosa, and lamina propria of the tuba uterina, but is less common in the appendix [5]. Most cases of ectopic decidua are related to normal pregnancy [5].

Ectopic decidua is generally asymptomatic and spontaneously regresses within 4–6 weeks after delivery [6]. Therefore, ectopic decidua is generally not treated [7]. However, appendiceal decidua can present as abdominal symptoms mimicking appendicitis [8]. In addition, it is necessary to treat cases of acute appendicitis, such as in our case.

The pathogenesis of ectopic decidual reactions is not yet fully understood. The most commonly accepted theory is metaplasia of the subcoelomic pluripotent mesenchymal cells due to the effect of progesterone. Another theory is “de novo” development of peritoneal decidual cells [9]. Furthermore, progesterone effects during pregnancy cause endometriotic foci to undergo marked stromal decidualization that resembles ectopic decidua [5]. Therefore, the pathogeneses of appendiceal decidua and decidualization of appendiceal endometriosis are different and must be clearly differentiated.

Both appendiceal decidua and decidualization of appendiceal endometriosis are diagnosed histopathologically. Appendiceal endometriosis can be seen in the muscular layer, seromuscular layer, and serosa of endometrial tissue, glandular tissue, and endometrial stroma [10]. Therefore, endometrial tissue is present in the stroma in patients with decidualization of appendiceal endometriosis, but is absent in those with appendiceal decidua.

There have been only four case reports of appendiceal decidua [6–8, 11], three of which included microscopic findings [6, 7, 11]. In all cases, the chief complaint was pain on the right side of the abdomen, and blood tests showed an elevated WBC count or increased CRP concentration. Ultrasonography and CT were performed in all cases. All patients had no fecaliths, and the appendiceal structures were confirmed in only one case. One report states that CT has high sensitivity and specificity in diagnosing appendicitis in pregnant patients [12]. However, the diagnostic accuracy of CT for appendiceal decidua may be low. Except for our patient, all patients ultimately underwent laparotomy. The use of laparoscopy provides better intraoperative visualization, less postoperative pain, shorter duration of hospitalization, quicker return to normal activities, and fewer abdominal wall complications compared with laparotomy [13].

There have been few reported cases of acute appendicitis caused by appendiceal decidua, and the mechanism of appendicitis has not been clarified. Decidualization of appendiceal endometriosis has been reported to occur due to increased luminal pressure, mucosal ischemia, bacterial proliferation, and inflammation [14]. Therefore, appendiceal decidua may have a similar mechanism to that of appendiceal endometriosis, leading to acute appendicitis.

Appendiceal decidua is an extremely rare disease that occurs in pregnant patients and is very difficult to diagnose on preoperative imaging studies. However, appendiceal decidua can lead to acute appendicitis, a diagnosis that may have to be made based on other findings, such as physical examination and blood tests. Therefore, when treating pregnant patients with suspected acute appendicitis, it is necessary to consider appendiceal decidua as a differential diagnosis.

## Data Availability

The data are available from the corresponding author upon reasonable request.
